# Porosity of Molecularly Imprinted Polymers Investigated by ^129^Xe NMR Spectroscopy

**DOI:** 10.1021/acsapm.2c01084

**Published:** 2022-11-04

**Authors:** Matteo Boventi, Michele Mauri, Kerstin Golker, Jesper G. Wiklander, Ian A. Nicholls, Roberto Simonutti

**Affiliations:** † Department of Materials Science, Università degli Studi di Milano-Bicocca, Via R. Cozzi 55, 20125, Milano, Italy; ‡ Linnaeus University Centre for Biomaterials Chemistry, Bioorganic and Biophysical Chemistry Laboratory, Department of Chemistry and Biomedical Sciences, Linnaeus University, SE-391 82 Kalmar, Sweden

**Keywords:** xenon NMR, time domain NMR, cross-linking, molecular imprinting, templated polymers

## Abstract

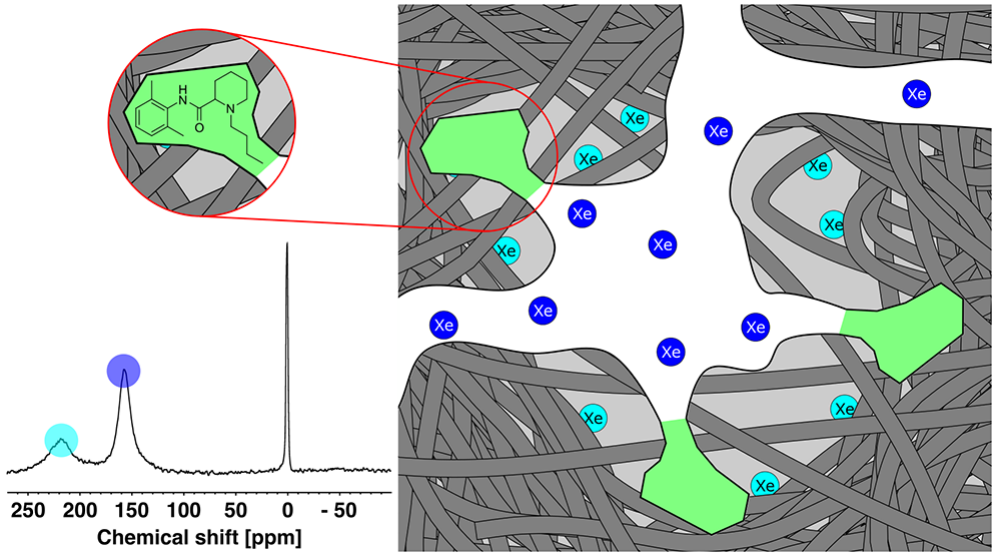

Molecularly imprinted polymers (MIPs) display intriguing recognition properties and can be used as sensor recognition elements or in separation. In this work, we investigated the formation of hierarchical porosity of compositionally varied MIPs using ^129^Xe Nuclear Magnetic Resonance (NMR) and ^1^H Time Domain Nuclear Magnetic Resonance (TD-NMR). Variable temperature ^129^Xe NMR established the morphological variation with respect to the degree of cross-linking, supported by ^1^H TD-NMR determination of polymer chain mobility. Together, the results indicate that a high degree of cross-linking stabilizes the porous structure: highly cross-linked samples display a significant amount of accessible mesopores that instead collapse in less structured polymers. No significant differences can be detected due to the presence of templated pores in molecularly imprinted polymers: in the dry state, these specific shapes are too small to accommodate xenon atoms, which, instead, probe higher levels in the porous structure, allowing their study in detail. Additional resonances at a high chemical shift are detected in the ^129^Xe NMR spectra. Even though their chemical shifts are compatible with xenon dissolved in bulk polymers, variable temperature experiments rule out this possibility. The combination of ^129^Xe and TD-NMR data allows attribution of these resonances to softer superficial regions probed by xenon in the NMR time scale. This can contribute to the understanding of the surface dynamics of polymers.

## Introduction

Micropores are an ideal and extremely tunable environment for catalysis, gas separation, gas storage, and supercapacitance. Pore size, shape, and decoration determines the performance of each process by precisely tuning both translational motion and internal configuration of the adsorbed molecule.^1^ This fueled the development of a plurality of materials such as zeolites, porous silica and alumina, metal organic frameworks (MOFs), and polymers of intrinsic microporosity (PIMs). The most relevant operational limit is the accessibility of the carefully tailored nanostructures to the environment. An elegant and effective solution to facilitate the exchange of molecules and ions to the active sites is the fabrication of hierarchical materials,^2^ where mesopore channels or exfoliated layers provide easy access to micropores hosted on their own walls without qualitatively affecting properties such as catalytic potential.^3^ The synthesis of these structures requires control on multiple scales and the optimization of many parameters. Monitoring by characterization techniques able to probe all of the different organization levels is thus mandatory. Such characterization tools should ideally be able to follow the structural evolution or the stability of the materials during operation, especially during demanding applications such as catalysis or energy storage.^4^ The micropores themselves can be extensively studied using nitrogen sorption interpreted by the Brunauer, Emmet, and Teller (BET) and the Barrett, Joyner, and Halenda (BJH) models; intrusion porometry; microscopy; and gravimetric methods, supported by computational simulations. The study of micropore accessibility is instead less definite and often reliant on spectroscopic methods that require sample transparency^5^ or on evaluating the reaction of molecules within a catalyst,^6^ an effective method that cannot be extended to noncatalytical materials. The study is further complicated when the hierarchical structures are comprised of soft matter, such as polymers or biomolecules, where, unlike rigid structures, it is also possible for probe molecules to competitively diffuse within the bulk as well as in the preformed channels. This is the case for many relevant biological systems such as blood purification in kidneys and in many bioinspired or biomimicking systems such as soft scaffolds for tissue regeneration or sensors for the detection of active molecules.

Molecular imprinting is a technique used to create porous polymers with predetermined recognition properties, which can be used in various applications due to their generally high physical and chemical stability as opposed to less stable biomacromolecules. To obtain a molecularly imprinted polymer via a noncovalent strategy, one must use monomers and a template in the prepolymerization mixture, which are capable of interacting with each other via electrostatic and/or van der Waals forces. The influence of these interactions on the final properties of molecularly imprinted polymers has been widely studied and reported in the literature.^7−12^ The recognition properties inevitably involve the analyte reaching the templated pores. A study regarding the effect of cross-linking in mesopore formation was previously performed by analyzing a series of methacrylic acid (MAA)-ethylene glycol dimethacrylate (EGDMA) copolymers synthesized using the local anesthetic bupivacaine as a template.^13^ By substituting one equivalent of EGDMA with two equivalents of methyl methacrylate (MMA), a series of samples with different degrees of cross-linking but equal numbers of sites capable of binding to the template was obtained while maintaining the concentration of heteroatoms involved in the binding. Molecular dynamics simulations indicated that a decrease in cross-linking density does not significantly decrease the hydrogen bonding interactions between MAA and the template in the prepolymerization mixture. Furthermore, nitrogen adsorption studies have shown that the porosity of the resulting polymers dictates the degree of interaction with the template. The decrease in binding capacity may be attributed to changes in polymer morphology arising from the lower degree of cross-linking, in particular decreased surface area and pore volume. Building on previous work, we used these well-characterized samples as a model system to ascertain the possibility of exploring the nature of the pore network by NMR, and to better understand the mechanism of adsorption in these particular systems. We used ^129^Xe NMR, exploiting the high sensitivity of Xe gas to the surrounding environment, to obtain detailed information about the porous structure of the polymers and the relationship between the degree of cross-linking and template recognition. In ^129^Xe NMR, differences in chemical shift are seldom influenced by interatomic connectivity, but rather by the interaction of the gas with the material, mediated by its motion within the millisecond time scale of the NMR experiment. Briefly, if one excludes the presence of highly charged cations and paramagnetic species, the chemical shift of xenon depends on its confinement: the stronger the confinement, the higher the chemical shift. A wide variety of models have been developed for interpreting the rich information contained in xenon spectra.^14^ A very important model was proposed by Terskikh et al. in 1993.^15^ According to this model, the chemical shift of xenon in mesopores results from a dynamic adsorption equilibrium between the “free” gas in the middle of the pore and the gas adsorbed on the pore walls. Even though this model is formally valid only for mesoporous silica, its application has been extended to a wide variety of porous materials.^16−19^ Xenon is not only able to travel within porous systems but also to diffuse in many polymers, providing a unique probe for systems such as porous organic materials.^20^ Since the pioneering works on ^129^Xe NMR in rubbery polymers,^21−26^ it has been shown that xenon is able to dissolve into the polymer bulk, experiencing a liquid-like environment and giving rise to characteristic NMR signals at high chemical shift values, usually between 180 and 220 ppm at room temperature, due to solute–solvent interactions. Given that the effectiveness of a MIP depends also on the chemical nature of the polymer matrix,^27^ the ^129^Xe NMR technique, which is sensitive to both chemistry and morphology, is an ideal method for characterizing such systems, where none of the organization levels in the hierarchy is periodic and where the pores themselves are always highly disperse.

## Experimental Section

### Polymer Synthesis

A series of four molecularly imprinted copolymers (MIP 1–4) was obtained by using bupivacaine as a templating agent, as described previously.^13^ The degree of cross-linking was varied by substituting one equivalent of EGDMA with two equivalents of MMA without changing the amounts of the other components. This approach allowed modifying the cross-linking density without altering either the number of heteroatoms able to form hydrogen bonds or the number of alkene functionalities. This is a crucial and often overlooked step in the preparation of nanostructured materials: hydrogen bond interactions in the prepolymerization mixture determine the final recognition properties of the polymers. The compositions of the prepolymerization mixtures are indicated in Table S1. From MIP 1 to MIP 4, the amount of cross-linker is decreased. A further decrease of cross-linking density, not reported in this paper, produces gel-like homogeneous materials with no significant porosity, as described previously.^13^

Briefly, the prepolymerization mixtures were prepared by dissolving appropriate amounts of (*R*,*S*)-bupivacaine (free base), MAA, MMA, EGDMA, and AIBN in toluene, in 100 mL KIMAX test tubes. The mixtures were sonicated to dissolve the AIBN, thereafter cooled on ice for 20 min followed by being purged with nitrogen for 20 min to remove dissolved oxygen. The polymerization was initiated by a UV source (Model UVGL-58, Upland, USA, 365 nm) and allowed to proceed for 24 h at 8 °C. The resultant polymer monoliths were manually ground, wet-sieved, and repeatedly sedimented, resulting in a final particle size of 25–63 μm. The template was removed by rigorous washing with acidic and alkaline solutions and the polymers were then dried at 60 °C for 24 h. Nonimprinted reference polymers were prepared similarly though in the absence of bupivacaine (REF 1–4).

### DSC

Calorimetric studies were performed using a DSC-1 system (Mettler Toledo). Around 5 mg of polymer were used for each experiment and placed in 40 μL Al crucibles with pierced lids. Experiments started with a temperature ramp between −120 and 25 °C and back to −120 °C. Then, samples were heated to 150 °C and cooled again to −120 °C. A final ramp to 150 °C was performed. For each ramp, the heating/cooling rate was 10 °C/min, and samples were kept under a nitrogen flow (80 mL/min) for the whole duration of the experiment.

### ^129^Xe NMR

Samples for ^129^Xe NMR were prepared as follows. Powder polymer samples (between 170 mg and 190 mg) were placed in thick wall Pyrex glass tubes (10 mm outer diameter, 8 mm inner diameter) and degassed directly under a dynamic vacuum (6.0 × 10^–2^ Torr) by connecting the tube to a Schlenk line. Several freeze–thaw cycles were performed, and the tubes were then left overnight under a vacuum to remove any residual traces of gas or water.

Xenon gas was quantitated by inserting it into a section of the line with known volume from where it was transferred into the NMR tube and frozen with liquid nitrogen. Lastly, tubes were flame-sealed while keeping the gas frozen, making sure that the sealing region was free of any trace of sample which could decompose and contaminate the tube. Table 1 shows the details of sample preparation, along with previously reported BET and BJH data.^13^^129^Xe NMR spectra were collected on a Bruker Avance 500 spectrometer operating at a Larmor frequency of 500.13 MHz for ^1^H and 138.45 MHz for ^129^Xe, equipped with a 10 mm broadband probe. Relaxation delay was set to 5 s for all spectra and the chemical shift of free xenon gas was set to 0 ppm to be used as an internal reference. For each sample, spectra were acquired at 25 °C, 0 °C, −20 °C, and −40 °C. Temperature control within 0.1 °C was provided by a BVT3000 variable temperature unit and a liquid nitrogen evaporator.

**Table 1:** Description of Sample Preparation for ^129^Xe NMR, Measured BET Surface Areas, and BJH Pore Volumes

sample	mass (mg)	nominal Xe pressure (bar)	BET surface area (m^2^/g)	BJH pore volume (cm^3^/g)
MIP 1	0.172	4.8	315 ± 1.9	0.909
MIP 2	0.178	6.0	163.5 ± 0.4	0.630
MIP 3	0.186	6.0	51.6 ± 0.3	0.332
MIP 4	0.180	6.1	29.2 ± 0.2	0.211
REF 1	0.171	6.0	324 ± 1.7	0.870
REF 2	0.192	6.0	135.6 ± 0.5	0.551
REF 3	0.190	6.0	45.1 ± 0.2	0.281
REF 4	0.189	6.5	15.2 ± 0.3	0.061

### TD-NMR

TD-NMR analysis was performed using the same tubes previously prepared for ^129^Xe NMR. This was done to ensure that the samples were protected from water and oxygen during the measurements. Analyses were performed with a Bruker Minispec mq20 operating at a Larmor frequency of 19.65 MHz for ^1^H. Temperature was set to 30 °C and maintained within ±0.1 °C with a Bruker BVT3000 variable temperature unit. The 90° pulse duration was calibrated to 2.1 μs, and the recycle delay was set to 1 s. The selected pulse sequence contained the Magic Sandwich Echo refocusing block, followed by a 90° pulse prior to signal acquisition. This pulse sequence is optimized for rigid systems since it allows for quantitative signal detection even in the presence of a significant dead time,^28^ which is on the order of 20 μs for common low field NMR instruments. For each sample, data were fit using a bimodal function that separates mobile and rigid contributions quantitatively and with high reliability.^29^ Each experiment was performed in triplicate, and the average values are reported.

## Results and Discussion

### DSC

**Figure 1:**
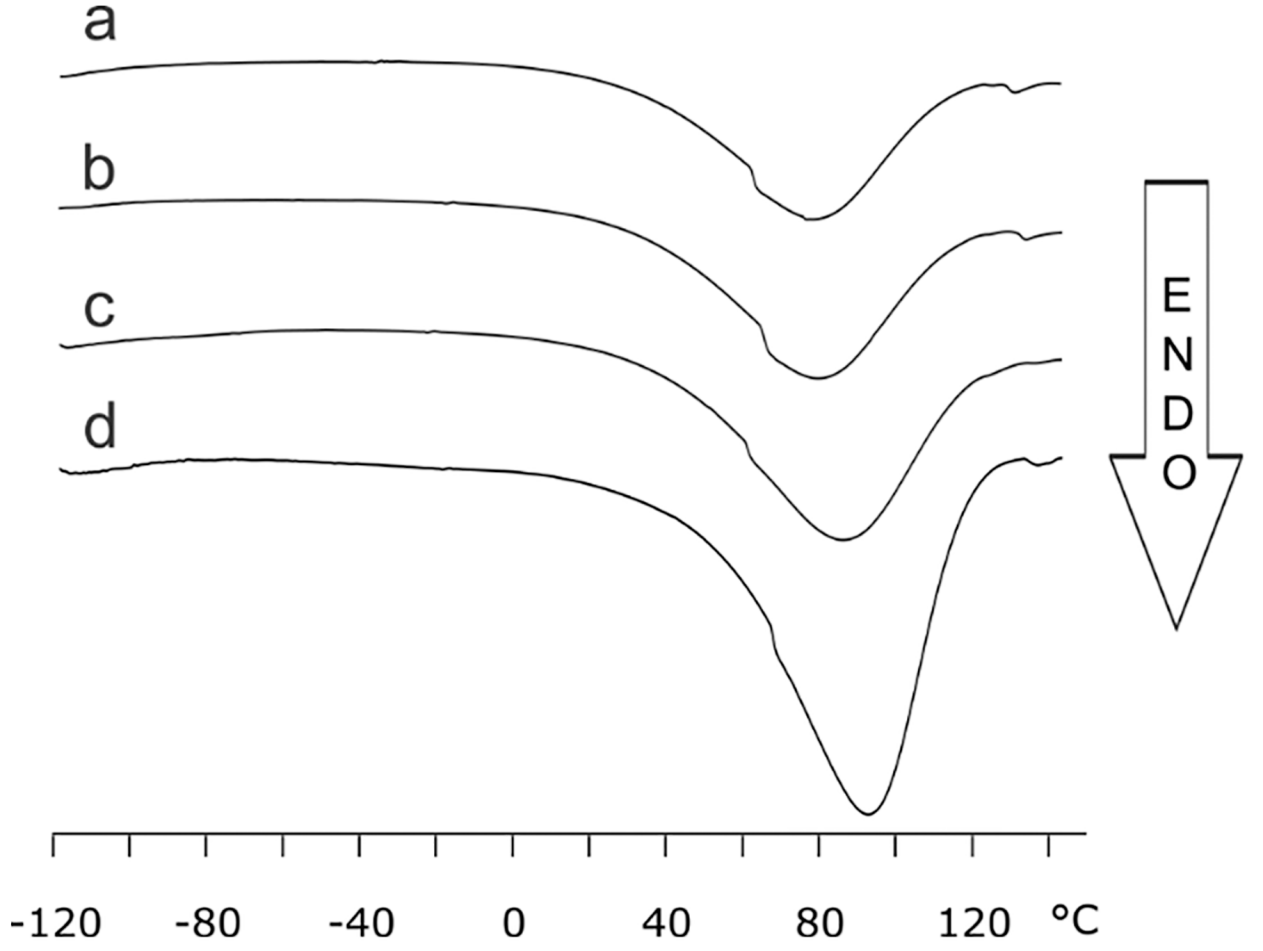
DSC thermograms of (a) MIP 4, (b) MIP 3, (c) MIP 2, and (d) MIP 1, showing the absence of any thermal event near the water melting point at 0 °C and the presence of broad events corresponding to water evaporation close to 100 °C.

The first heating cycle (from −120 to 25 °C and then back to −120 °C) was performed to detect any low temperature glass transition before analyzing the calorimetric behavior of the samples at higher temperatures, possibly changing their delicate structure. The corresponding curves did not show any significant features (Figure S1). On the contrary, the third ramp that runs from −120 °C up to 150 °C shows a broad endothermal peak close to 100 °C for all samples (Figure 1). This is associated with water evaporation, since by repeating one final ramp from 0 to 150 °C (Figure S1), only a vestigial peak appears at high temperatures (120 °C) corresponding to the removal of trace water in the matrix.

As further confirmation, by measuring the weight of DSC crucibles after the experiments, we detected a mass reduction of around 6–7% for all samples. By leaving the samples in air overnight, the weight reverted to about 99% of the starting value, confirming that this weight loss was due to water evaporation and not to polymer degradation or evaporation of other species such as residual monomers. Interestingly, the formation or melting of ice was never seen by DSC in the −120 to 25 °C range, indicating that water is sorbed within the bulk and does not consist of droplets within the pores. In detail, the water evaporation temperature is influenced by porosity: samples with lower cross-linking, and thus wider pores, display evaporation at lower temperatures and lower associated enthalpy (see Table S2). In the third heating ramp, performed after water removal, the calorimetric curves display no evidence of glass transition (Figure S1). This behavior is typical of hyper-cross-linked structures lacking long linear segments able to undergo concerted motions that characterize the glass transition.^1^ It was anticipated that differences in Δ*H* between the highly cross-linked, MIP 1 and REF 1, and less cross-linked, MIP 4 and REF 4, could be attributed to this phenomenon. Indeed, Table S1 shows that the cross-linker, EGDMA, even in the least cross-linked samples MIP 4 and REF 4, has a molar fraction close to 11.5%. This value is much higher than standard cross-linked systems, such as vulcanized rubber where the cross-linker is usually a few phr,^30^ indicating that all samples in the present paper can be considered hyper-cross-linked. Still, even this level of cross-linking is insufficient to form a stiff structure on the microscopic scale, that is instead formed in samples almost totally comprised of EGDMA.

**Figure 2:**
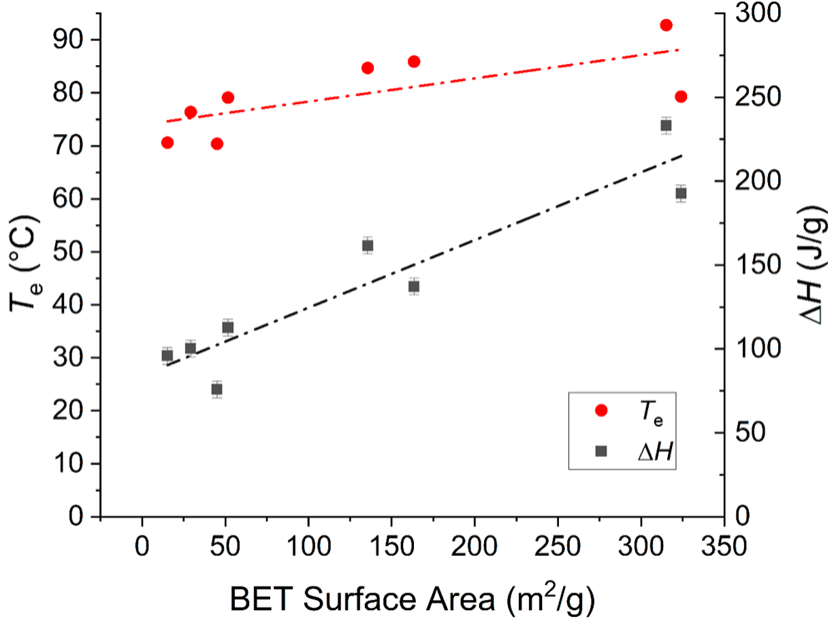
Plot of the calorimetric parameters of water evaporation as a function of the specific surface area of the MIP and REF samples.

A quantitative analysis of the DSC data in Table S2 is presented in Figure 2, where the temperature and enthalpy of the endothermal event associated with water evaporation are plotted for each sample as a function of the respective specific surface area, not considering the composition. The plot indicates a weak general trend toward higher evaporation enthalpy and higher peak temperature as the specific surface area is increased.

Together, the data indicate that water is sorbed within the polymers, and the amount of water and evaporation mechanism are partially mediated by the availability of the surface.

### TD-NMR

Since DSC curves do not provide any evidence of glass transition, ^1^H TD-NMR was performed to characterize the bulk materials and their mobility at the molecular level. This technique is particularly well-suited to measure the dynamic state of polymer chains,^31^ which is another factor that can influence the interaction with the template. Using a pulse sequence containing the Magic Sandwich Echo refocusing block allowed quantification of the amount of rigid and mobile fractions of the polymers and their corresponding effective transversal relaxation times (*T*_2_*). This information gives a more in-depth view of the mobility of the system compared to DSC, clearly distinguishing polymers below and above the *T*_g_ even on the microscopic scale. Using vacuum-dried and sealed samples ensured that the results are indicative of the inherent mobility of the polymer chains and not altered by the plasticizing effect of water.^32^

**Figure 3:**
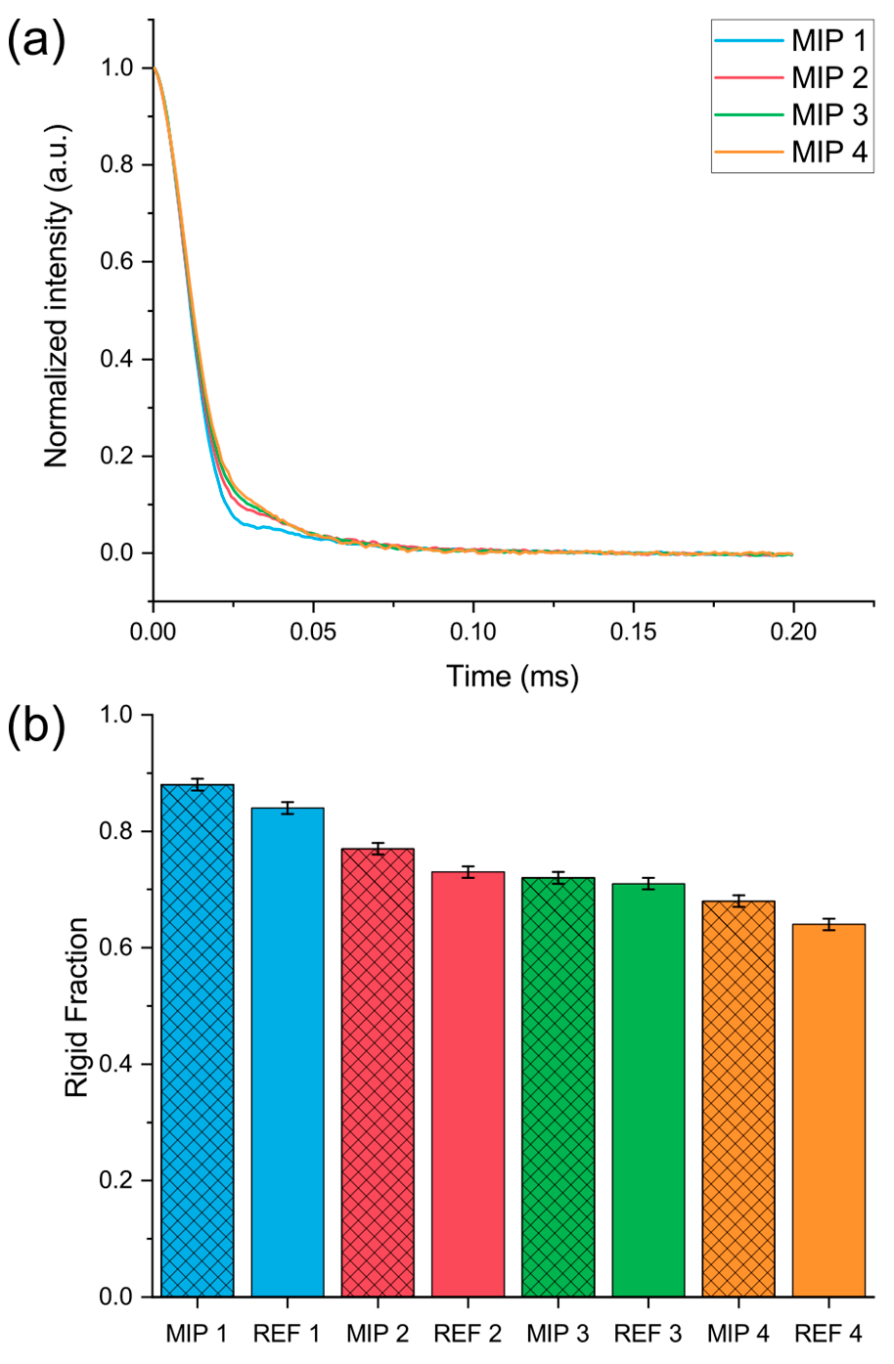
(a) Normalized FIDs of MIP samples derived from the Magic Sandwich Echo refocusing sequence, showing the prevalence of the fast relaxation associated with protons in the rigid phase. (b) Rigid fraction values resulting from a bimodal fitting of the FIDs.

Representative free induction decays (FIDs) for MIP samples are shown in Figure 3a, while similar looking FIDs of REF samples are represented in Figure S2. Despite the different degrees of cross-linking, each sample shows a fast decay of FID intensity, typical of materials with a relevant rigid fraction, followed by a small tail due to mobile fractions. A bimodal behavior was detected even for the most cross-linked materials, and thus, a bimodal function was used to fit the data (Figure S2). The rigid fraction values resulting from fitting of the FIDs are shown in Figure 3b. They confirm that all materials are predominantly rigid, with *R* values ranging from 0.88 for MIP 1 to 0.64 for REF 4. These values decrease with a decreasing degree of cross-linking, indicating relatively softer materials as more of the EGDMA in the prepolymerization mixture is substituted by MMA. Moreover, as expected for very rigid materials, the *T*_2_ values are very low, even for the mobile fractions (Table S3). By comparing MIP samples with the corresponding REF samples, it appears that the former show systematically higher rigid fraction values compared to the latter. To understand this trend, one must first analyze what happens during the synthesis of these materials and understand the mechanism behind the formation of molecularly imprinted polymers.^12^ Briefly, in noncovalent imprinting, the most important factor in determining the molecular recognition properties is the favorable formation of polyvalent complexes between growing polymer chains and template molecules. During the first stages of the synthesis, the oligomers change their conformation to maximize the interaction with the template, thereby forming relatively labile recognition sites. As the polymerization continues and cross-linking occurs, the polymers progressively lose their flexibility and the recognition sites become more and more stable. Thus, the presence of the template molecules influences not only the recognition properties of the final materials but also their conformation and mobility, which are determined during the synthetic process. As such, when relating MIP and REF samples, which have the same composition but are synthesized in the presence or absence of the template, one has to keep in mind that, for the former, the presence of the template strongly influences the growth of the polymer chain from a conformational point of view. The molecular mobilities of the resulting materials are different, even if only slightly, and this difference can be detected by TD-NMR. More specifically, the presence of the template molecule during polymer synthesis causes a slight decrease in the mobility of the protons of the resulting materials which translates to a slight increase of the rigid fraction values compared to the nonimprinted polymers. This effect, as seen in Figure 3b, is small but significant for the MIP 1 and REF 1 couple as well as for the MIP 4 and REF 4 couple and becomes less pronounced in MIP 2 and MIP 3. The effect is within the detection limits of TD-NMR, which has proven extremely sensitive in various situations where slight variations of the rigid fraction have been used to characterize a wide variety of materials for various applications.^29^

### ^129^Xe NMR

**Figure 4:**
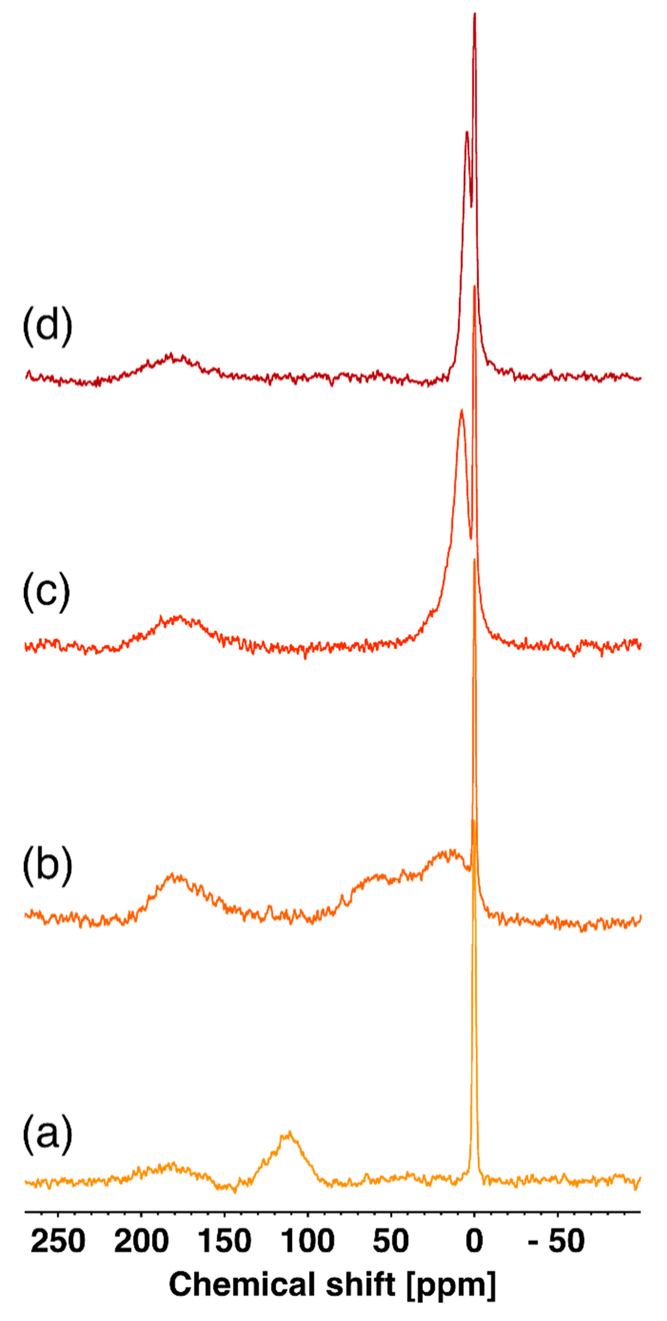
^129^Xe NMR spectra of (a) MIP 1, (b) MIP 2, (c) MIP 3, and (d) MIP 4 acquired at 25 °C.

The ^129^Xe NMR spectra at 25 °C of all MIP samples are shown in Figure 4. All spectra show a sharp and intense resonance associated with free gas outside the pore system, set to 0 ppm and used as an internal reference. In addition, in all spectra a broad peak can be seen roughly around 180 ppm. Peaks in this range are usually associated with xenon dissolved in the polymer bulk,^26^ but their exact nature will be discussed in detail below.

**Figure 5:**
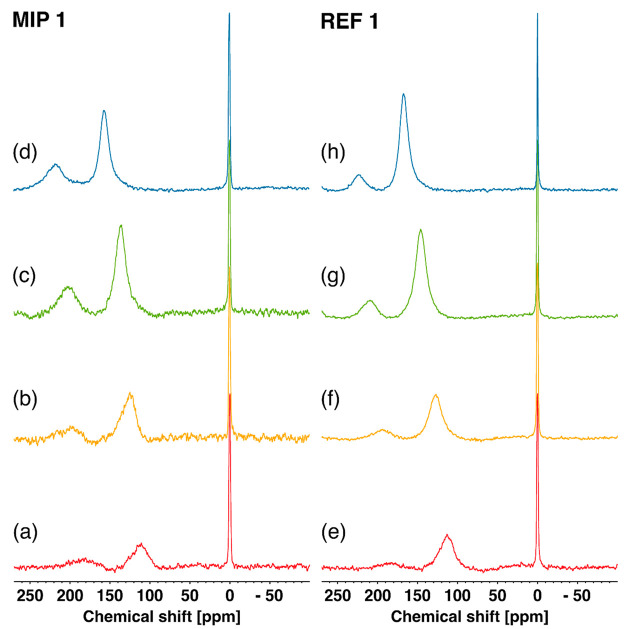
^129^Xe NMR spectra of samples MIP 1 and REF 1 acquired at (a, e) 25 °C, (b, f) 0 °C, (c, g) −20 °C, and (d, h) −40 °C.

The remaining resonances are descriptive of the pore structure, and for this reason their appearance varies significantly from one system to the other. The spectrum of MIP 1 shows a broad peak around 110 ppm, which is associated with a wide distribution of nonuniform and interconnected mesopores, as confirmed by variable temperature experiments (see Figure 5 below). MIP 2 displays an even broader, roughly bimodal resonance region between 0 and 80 ppm. This indicates the presence of different regions of communicating pores of widely different sizes, some of which are close enough to the surface to allow xenon atoms to exchange directly with the free gas outside. In contrast, MIP 3 exhibits a relatively sharp peak with roughly double the line width of the free gas and a chemical shift close to 10 ppm. This resonance results from xenon probing very large pores, where it is only slightly more constrained than in the free gas. Atomic motion in the environment within the NMR time scale (ms) causes a reduction of line width due to signal averaging. This trend is confirmed by the even sharper peak and lower chemical shift, 4.7 ppm, of MIP 4.

The spectra from the corresponding REF samples (Figure S4) are very similar, with only slightly different chemical shifts that can reflect the differences in specific surface area, which in some cases are significant since the preparation of cross-linked samples is generally difficult to control. MIP and corresponding REF samples, despite having been prepared in parallel with the same composition except for the presence of bupivacaine, sometimes display important differences in global pore volume (see Table 1).

The comparison of the spectra between paired samples with the closest specific surface (e.g., MIP 1 vs REF 1 or MIP 3 vs REF 3) also indicates no direct effect of the presence of bupivacaine during the polymerization steps. According to molecular modeling, the size of bupivacaine in the conformation that maximizes recognition is around 0.9 × 0.5 nm. The xenon atom diameter is close to 0.44 nm. However, acrylic polymer systems similar to those studied here have been observed to exhibit significant swelling behavior in the wet state.^10,11^ Thus, idealized pores in the dry state may be too small to accommodate a xenon atom: its diffusion is instead probing higher levels in the pore hierarchy. Under rebinding conditions, however, the effect of polymer swelling is anticipated to facilitate diffusion of bupivacaine molecules into the templated pores. This view is supported by the fact that MIP 1 sample still containing the template molecule presents a ^129^Xe NMR spectrum almost coincident with MIP 1, in which bupivacaine has been removed (Figure S5).

In xenon NMR, variable temperature experiments are quite valuable since the chemical shift and the intensity of the resonances are modulated by the dynamics of xenon atoms moving in the free space. For all MIP and REF samples, variable temperature experiments were performed. In Figure S8, the variable temperature spectra of the sample of intermediate porosity MIP 3 are depicted. At higher temperatures, a narrow peak, positioned near the gas reference, indicates a fast exchange that favors the larger pores (Figure S8a). As the temperature is decreased, the signal broadens and splits into several signals associated with different families of pores. Finally, at −40 °C (Figure S8d), xenon pools within a broad distribution of smaller mesopores. Note that in all of these processes, the signal around 180 ppm follows its own independent and roughly linear evolution of the chemical shift while always remaining broad.

**Figure 6:**
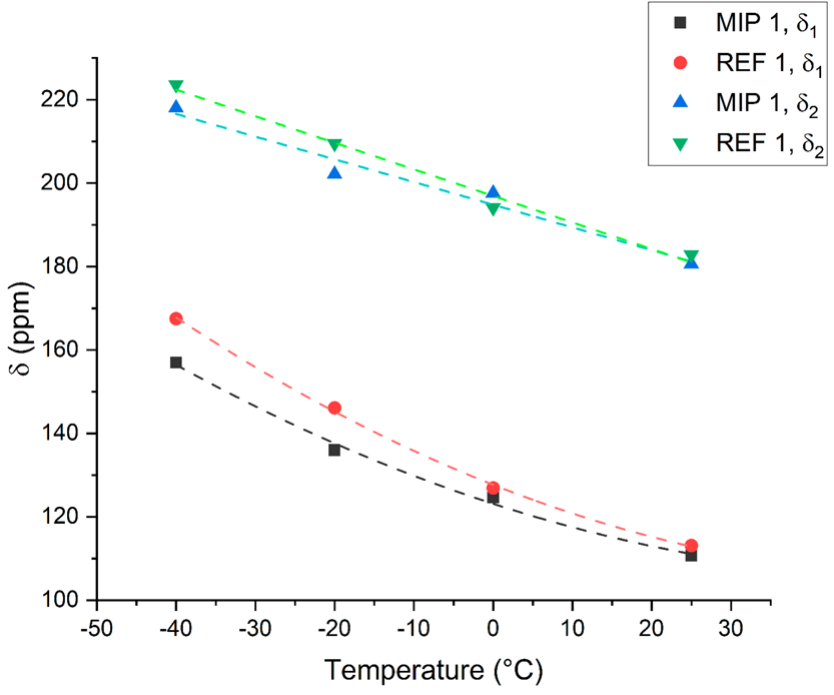
Temperature dependence of the chemical shifts of both Xe–sample interaction peaks in MIP 1 and REF 1. δ_1_ indicates the peak closest to the free gas, while δ_2_ indicates the broad peak at high chemical shift values. The dashed traces are visual aids for the δ_1_ peaks and linear fits for the δ_2_ peaks.

Other samples, reported extensively in Figures S6 to S11, present the same qualitative trends. This can be studied more precisely using the spectra for the most porous systems, MIP 1 and REF 1, reported in Figure 5. It can be seen that both samples show an analogous behavior in a wide range of temperatures, further confirming that xenon cannot detect any differences due to the presence of templated pores. For these samples, it was possible to detect and assign all resonances unambiguously at all temperatures, and thus, the temperature dependences of the two peaks in the studied range were determined. The results are shown in Figure 6. The dependence of the intermediate peak (called δ_1_) with temperature is nonlinear, roughly following the expected temperature dependence for xenon diffusing in mesopores,^15,33^ confirming that this resonance is attributable to a wide distribution of nonuniform and connected mesopores. Another important observation is that for the peak assigned to xenon atoms most closely associated with the polymer, δ_2_, the shift follows a linear trend over the observed range which spans 65 °C, despite the noise due to the broad line width of the signals. Linear fits of these data (Figure S13) returned a temperature dependence of −0.64 ppm/°C for MIP 1 and of −0.54 ppm/°C for REF 1. Miller et al. reported a temperature dependence around −0.3 ppm/°C for xenon dissolved in a wide variety of bulk polymers,^26^ and similar values were reported by Morgado et al. for a wide variety of linear and branched alkanes and cycloalkanes.^34^ The values obtained for the δ_2_ peaks of samples MIP 1 and REF 1 are much larger, indicating that xenon is experiencing a radically different environment in these samples. Since the δ_2_ peak followed a roughly linear trend even for the polymers with lower porosities, the temperature dependence of this peak was determined for all of the samples. The results are summarized in Table 2. The obtained values are similar and always lower than −0.3 ppm/°C, indicating that this signal represents a somewhat analogous environment in each sample.

**Table 2:** Chemical Shifts of the Peaks Assigned to Xenon Inside the Pores (δ_1_) and in the Pseudobulk (δ_2_) and the Temperature Dependence of δ_2_

Sample	δ_1_ (ppm) 25 °C	δ_2_ (ppm) 25 °C	δ_1_ (ppm) −40 °C	δ_2_ (ppm) −40 °C	*T* dep δ_2_ (ppm/°C)
MIP 1	110.7	180.6	157.0	218.0	–0.57
MIP 2	2.5–95 (broad)	178.0	144.9	228.6	–0.76
MIP 3	7.8	177.7	90.3	221.1	–0.71
MIP 4	4.7	179.3	broad	undetectable	–0.61
REF 1	113.1	182.8	167.5	223.6	–0.64
REF 2	10.9	185.7	135.4	225.2	–0.64
REF 3	7.7	188.5	92.4	220.5	–0.44
REF 4	undetectable	187.7	6.5	226.3	–0.65

To further analyze the nature of the peak associated with stronger adsorption, we decided to investigate MIP 1 at lower nominal xenon pressure, 1.4 bar. The resulting variable temperature spectra are shown in Figure S12. At 25 °C, the xenon atoms in the mesoporous structure generate a broad and weak resonance at around 108 ppm, analogous to that observed at higher pressure (Figure 4). The δ_2_ peak, instead, cannot be observed, possibly due to the extreme broadening combined with a low signal-to-noise ratio. At 0 °C, the very broad δ_2_ signal becomes slightly visible, while the other peak shifts downfield and becomes sharper and more intense. By further lowering the temperature, both signals shift downfield and gain intensity relative to the free gas peak. The evidence from Figure S12 suggests a strong dependence of signal δ_2_ on xenon pressure, indicating a possible relation of this signal with the surface of the pores.

Interestingly, the intensity of the δ_2_ resonance does not seem to be dependent on the amount of sample present in each tube, which is roughly constant. For example, the signal in MIP 1 is almost invisible, and it is instead very intense in MIP 2. This is another important indication that this signal is not simply due to xenon dissolution in the bulk: in that case, the signal intensity should be proportional to the amount of sample. We also note that the water absorption is instead proportional to the mass, around 6% w/w for all samples. This is a high value relative to bulk poly(methyl methacrylate), which has a hygroscopicity of around 2%, but in line with the hyper-cross-linked nature of the samples, which reduces the capability of the polymer to fill all the local voids. The compatibility of these polymers with water is facilitated by the presence of MMA/EGDMA and of the even more polar methacrylic acid. This high adsorption of a polar molecule, such as water in the bulk, suggests that it is a hydrophilic environment, not prone to absorbing the lipophilic apolar Xe gas. Thus, the interaction of xenon with the MIP does not take place by the same mechanism, and possibly not even in the same sites. Moreover, TD-NMR indicated that the samples are mostly rigid, that is, below the glass transition temperature.

A possible identification of the xenon sorption sites is provided by the work of De Gennes regarding the glass transition temperature of polymer surfaces,^35^ proposing a gradual depression of the glass transition temperature close to the surface, confirmed experimentally on thin films.^36,37^ While many papers still discuss this open topic, both on polymers and on the polymer coating of nanoparticles,^38^ very few tackle its applicability on cross-linked or hyper-cross-linked systems.^39^ Mobility measurements performed by TD-NMR always indicate the presence of a mobile fraction. It does not seem related to the specific surface, since increases of surface area do not correspond to an increase of mobile fraction, but since TD-NMR provides an evaluation that is mediated throughout the sample, we cannot exclude that the surface is indeed softer and capable of Xe adsorption. We believe that the δ_2_ signal is associated with rough and soft zones on the surface of these materials, where xenon strongly interacts with the polymer chains, leading to chemical shifts comparable to those of Xe dissolved in bulk polymers. According to this model, we can consider the δ_2_ peak as a *pseudobulk* signal since the chemical shift is similar to that in bulk polymers, but its underlying origin is different.

The δ_1_ chemical shifts and the corresponding trends of pore size are in line with the current understanding of the mechanism of pore formation within MAA-EGDMA/MMA copolymers in the presence of a porogen such as toluene.^11,13^ As the polymerization proceeds, the polymer is formed around solvent droplets, as in the formation of PS-DVB polymers. In that widely studied case, increasing the amount of cross-linker also increases the pore surface and provides better control over pore size.^40^ This method can produce strong local inhomogeneities, thus in order to obtain homogeneous porosity it is more convenient to use “Davankov-type resins”, where preformed polymer chains are directly cross-linked.^41^ It is thus expected that, during copolymer synthesis, the microphase separation will produce a wide variety of collapsed pores in samples with low degrees of cross-linking. This was confirmed by the specific surface data in Table 1, while a more in-depth analysis of BET and BJH data indicated that the surface areas and pore volumes are actually integrated over a wide distribution of porosity sizes, where the overall pore volume and the average fraction of smaller pores decreased with decreasing cross-linking density.^13^ In fact, the adsorption porometry measurements did not simply indicate that the highest cross-linked sample has smaller pores while the less cross-linked have larger pores. On the contrary, all samples displayed pores in all ranges between 2 and 200 nm. A much higher level of detail is instead provided by ^129^Xe NMR, thanks to the dynamic nature of this technique. The highly cross-linked samples MIP 1 and REF 1 are characterized by a wide distribution of nonuniform and connected mesopores, which are indicated by the wide resonance around 100 ppm. In samples MIP 4 and REF 4, signals close to the free gas peak correspond to xenon probing very large void spaces and exchanging with the free gas. By lowering the temperature, these signals progressively become broader and even disappear in the case of MIP 4. At low temperatures, the adsorption of xenon on the pore walls is favored, and the nuclei start to probe a very wide distribution of pores, giving rise to extremely broad peaks close to the baseline. This interpretation is consistent with the expected structural collapse due to an insufficient degree of cross-linking. The porosities of samples MIP 2/REF 2 and MIP 3/REF 3 are intermediate between the stable porous structure of samples MIP 1/REF 1 and the collapsed structure of MIP 4/REF 4. For samples MIP 2 and REF 2, the presence of a broad distribution of pores of widely different sizes is evidenced by the spectra at 25 °C. By lowering the temperature, the spectra become more similar to those of MIP 1/REF 1. This indicates that only a certain part of the porous structure is constituted by well-defined and stable mesopores, while the remaining part is made up of collapsed pores. Samples MIP 3/REF 3 show a somewhat analogous behavior, but the collapsed pores are even more predominant, and the smaller and stable ones can only be detected at low temperatures.

**Figure 7:**
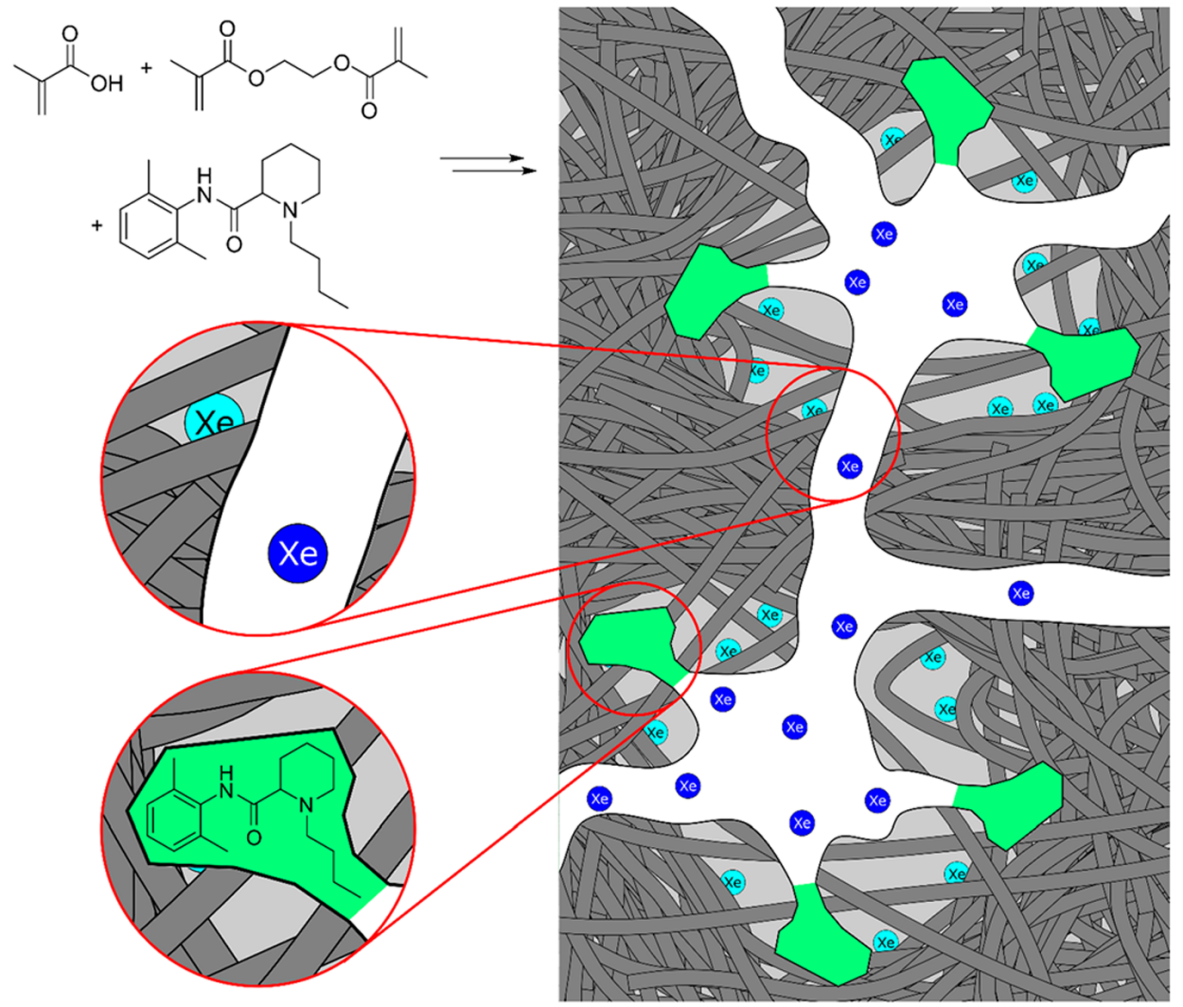
Schematic representation of the structure of MIP 1 as detected by ^129^Xe NMR. Xenon atoms in blue are confined to the porous structure of the polymer, while xenon atoms in light blue are adsorbed on the polymer surface. Templated pores that are spatially and chemically complementary to the imprinted molecule are represented in green.

Finally, to explain the ^129^Xe NMR data, we propose a simple model for MIP 1, presented in Figure 7. Xenon atoms in blue probe the porous structure of the polymer, which is kept stable by the high degree of cross-linking. They are associated with the intermediate resonance, which indicates the presence of a broad distribution of mesopores inside the polymer structure. As stated previously, ^129^Xe NMR does not show any differences due to the presence of templated pores in dry state MIP samples because the pores are too small for xenon. Light blue xenon atoms are associated with the so-called pseudobulk signal. They interact with all accessible bulk polymer surfaces, and this strongly influences xenon’s chemical shift, relative to xenon atoms in the mesoporous structure.

## Conclusions

In this study, the influence on polymer morphology of the degree of cross-linking and of the presence of template has been examined in a series of bupivacaine molecularly imprinted polymers using TD-NMR and ^129^Xe NMR. Following the differences in polymer composition, significant differences in polymer microstructure and rigidity were observed using TD-NMR and were correlated to polymer surface area and pore size distribution. ^129^Xe NMR detected two different populations of xenon atoms that do not significantly exchange during the NMR experiment time and whose separate evolution can be used to monitor the MIP material both at the compositional and at the morphological level.

Higher cross-linking consistently helps the formation and stabilization of extensive and interconnected pores. Bupivacaine shaped imprints on the surface are too small and not specific enough to trap xenon that detects them as crenellations on the surface.

In the spectral region associated with higher confinement, broad signals were detected with a chemical shift highly dependent on temperature. This observation is consistent with a complex interaction where xenon is strongly associated and possibly dissolved into a surface layer that is looser than the rest of the polymer matrix while still maintaining the structural rigidity imparted by hyper cross-linking: this is a direct proof that highly cross-linked polymer systems present a *T*_g_ depression similar to the one postulated by De Gennes for surfaces of linear polymers.

These findings demonstrate the ability of Xe NMR to elucidate hierarchical systems even in the case of soft matter, meaning that not only MIPs but also many other systems, like polymeric foams, colloids, and core–shell particles can be characterized following the same approach.

## Supporting Information Available

Composition of the prepolymerization mixtures of the materials; DSC data and discussion; fitting procedure and results of MSE TD-NMR experiments; ^129^Xe NMR spectra of all REF samples at room temperature; room temperature ^129^Xe NMR spectrum of sample MIP 1 in the presence of bupivacaine; variable temperature ^129^Xe NMR of all MIP and REF samples not presented in the main text; temperature-dependent plot of the chemical shift of the δ_2_ signal (PDF)
