# Supplementary material for: Porosity of Molecularly Imprinted Polymers Investigated by 129Xe NMR Spectroscopy
Source: ACS Appl Polym Mater. 2022 Nov 4;4(12):8740–9. doi: 10.1021/acsapm.2c01084 (PMC9745730; doi:10.1021/acsapm.2c01084)
Supplement: Supplementary file 1 — ap2c01084_si_001.pdf [file ap2c01084_si_001.pdf]

Supporting Information

# Porosity of Molecularly Imprinted Polymers investigated by $^{129}$Xe NMR spectroscopy

*Matteo Boventi,[a] Michele Mauri,[a] Kerstin Golker,[b] Jesper G. Wiklander,[b] Ian A. Nicholls,[b] Roberto Simonutti[a]**

[a]) Department of Materials Science, Università degli Studi di Milano-Bicocca, Via R. Cozzi 55, 20125, Milano, Italy

[b]) Linnaeus University Centre for Biomaterials Chemistry, Bioorganic and Biophysical Chemistry Laboratory, Department of Chemistry and Biomedical Sciences, Linnaeus University, SE-391 82 Kalmar, Sweden

Corresponding Author email: roberto.simonutti@unimib.it

KEYWORDS. Xenon NMR, Time domain NMR, crosslinking, molecular imprinting, templated polymers.

CONTENT

1. Composition of the molecularly imprinted polymers

2. DSC data and discussion

3. Fitting procedure and results of MSE TD-NMR experiments

4. $^{129}$Xe NMR spectra of all REF samples at room temperature

5. Room temperature $^{129}$Xe NMR spectrum of sample MIP 1 in the presence of bupivacaine

6. Variable temperature $^{129}$Xe NMR all MIP and REF samples not presented in the main text, and temperature dependent plot of the chemical shift of the $\delta_2$ signal

## 1. Composition of the molecularly imprinted polymers

**Table S1.** Composition of the MIP materials and their REF counterparts.

| Sample | Bupivacaine | MAA | MMA | EGDMA | AIBN | Toluene | Molar ratio |
|---|---|---|---|---|---|---|---|
| **MIP 1** | 1.39 | 20.1 | 0 | 82.3 | 2.40 | 259.4 | 1:14:0:59:187 |
| **MIP 2** | 1.39 | 20.1 | 40.0 | 57.4 | 2.27 | 259.4 | 1:14:29:41:187 |
| **MIP 3** | 1.39 | 20.1 | 80.1 | 37.4 | 2.27 | 259.4 | 1:14:58:27:187 |
| **MIP 4** | 1.39 | 20.1 | 120.1 | 17.4 | 2.27 | 259.4 | 1:14:86:13:187 |
| **REF 1** | 0 | 20.1 | 0 | 82.3 | 2.40 | 259.4 | 0:14:0:59:187 |
| **REF 2** | 0 | 20.1 | 40.0 | 57.4 | 2.27 | 259.4 | 0:14:29:41:187 |
| **REF 3** | 0 | 20.1 | 80.1 | 37.4 | 2.27 | 259.4 | 0:14:58:27:187 |
| **REF 4** | 0 | 20.1 | 120.1 | 17.4 | 2.27 | 259.4 | 0:14:86:13:187 |

*Amounts are in mmol and molar ratio is bupivacaine:MAA:MMA:EGDMA:toluene.*

## 2. DSC data and discussion

The presented trace is representative of all experiments. It is apparent that during the first heating ramp from -120 to 25 ˚C no thermal events can be detected. The middle heating ramp from -100 ˚C to 120 ˚C is discussed in the main text for all samples. On the final heating ramp, a vestigial peak appears at 120 ˚C corresponding to the removal of trace water in the matrix.

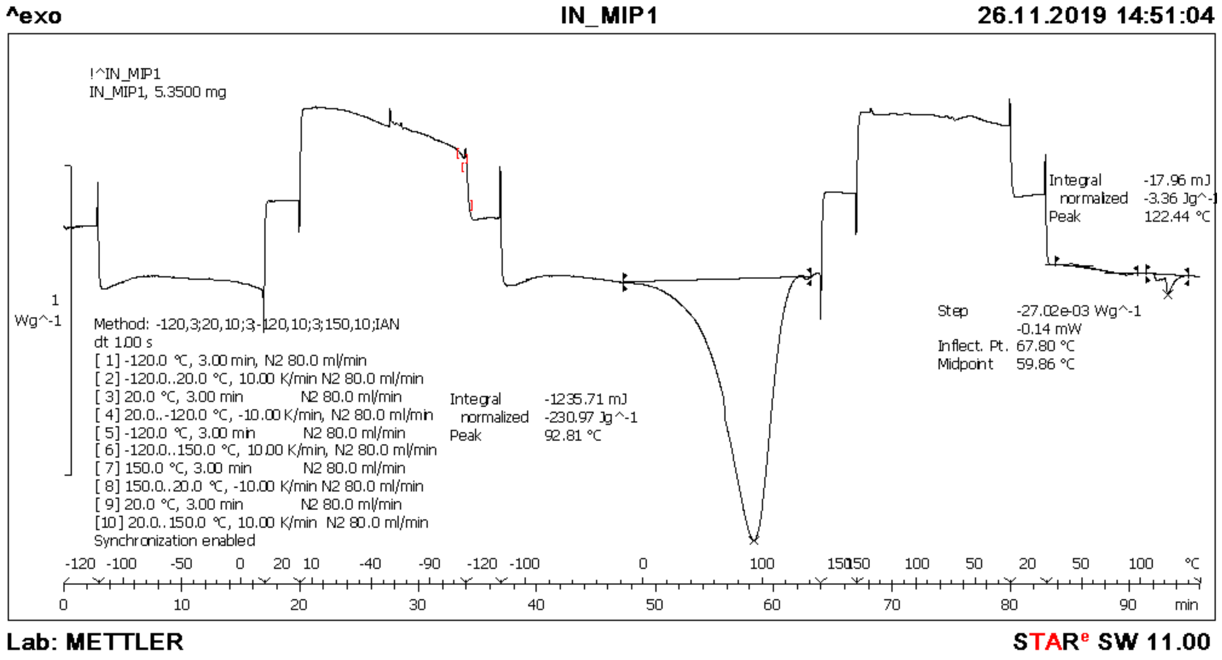


**Figure S1.** Full thermogram of sample MIP 1, comprising several heating and cooling ramps.

**Table S2.** Water evaporation data of the samples.

| Sample | Water evaporation temperature (˚C) | Δ*H* (J/g) |
|---|---|---|
| **MIP 1** | 92.8 | 233 |
| **MIP 2** | 85.9 | 137 |
| **MIP 3** | 79.1 | 113 |
| **MIP 4** | 76.4 | 100 |
| **REF 1** | 79.3 | 193 |
| **REF 2** | 84.7 | 162 |
| **REF 3** | 70.4 | 76 |
| **REF 4** | 70.6 | 96 |

## 3. Fitting procedure and results of MSE TD-NMR experiments

Normalized free-induction decays (FIDs) obtained from Magic Sandwich Echo (MSE) experiments were fit to the following bimodal function

$$I(t) = Re^{-\left(\frac{t}{T_{2,r}^{*}}\right)^{2}} + (1-R)e^{-\left(\frac{t}{T_{2,m}^{*}}\right)^{2}}$$

where *R* represents the percentage of the rigid fraction, $T_{2,\,r}$* being the apparent relaxation time of the gaussian component associated to the rigid fraction, and $T_{2,\,m}$* is the spin-spin relaxation time of the mobile part.

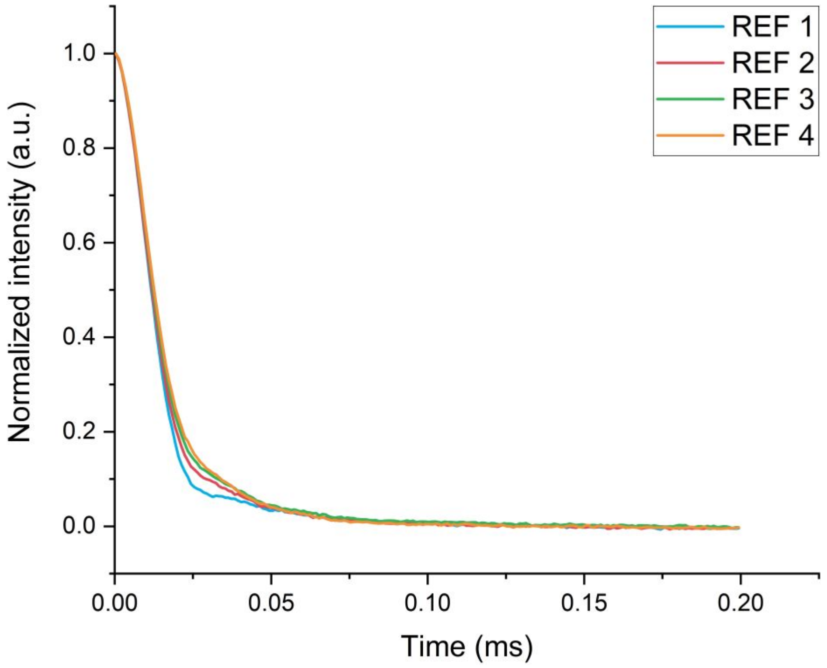


**Figure S2.** Normalized FIDs of REF samples derived from the Magic Sandwich Echo refocusing sequence.

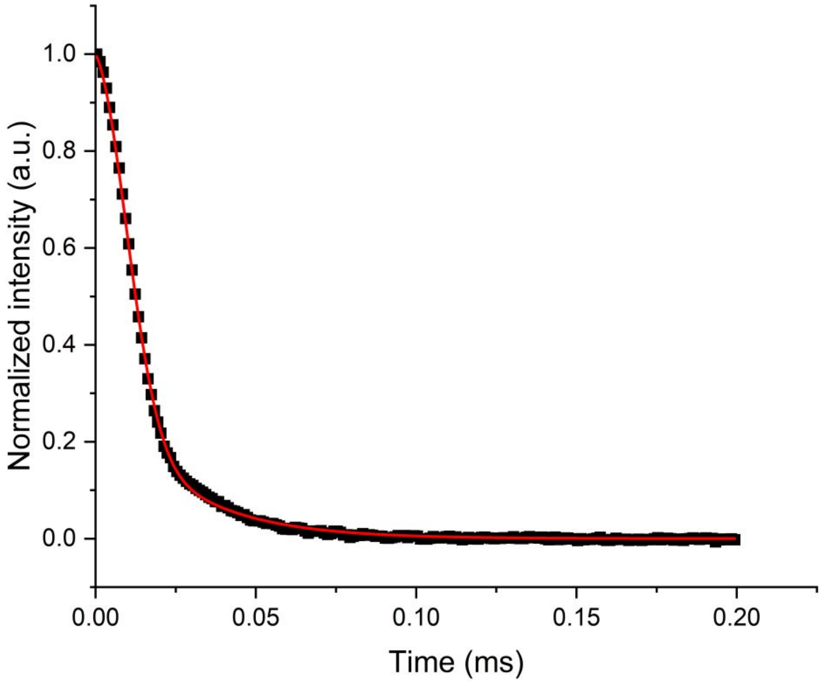


**Figure S3.** Fitting of the MSE FID of sample MIP 4.

**Table S3.** Parameters derived by fitting the bimodal function to normalized MSE FIDs.

| Sample | $R$ | $T_{2,\,r}$* (μs) | $T_{2,\,m}$* (μs) |
|---|---|---|---|
| MIP 1 | 0.88 | 13 | 35 |
| REF 1 | 0.84 | 13 | 32 |
| MIP 2 | 0.77 | 13 | 29 |
| REF 2 | 0.73 | 13 | 26 |
| MIP 3 | 0.72 | 14 | 26 |
| REF 3 | 0.71 | 14 | 29 |
| MIP 4 | 0.68 | 14 | 24 |
| REF 4 | 0.64 | 14 | 25 |

## 4. $^{129}$Xe NMR spectra of all REF samples at room temperature.

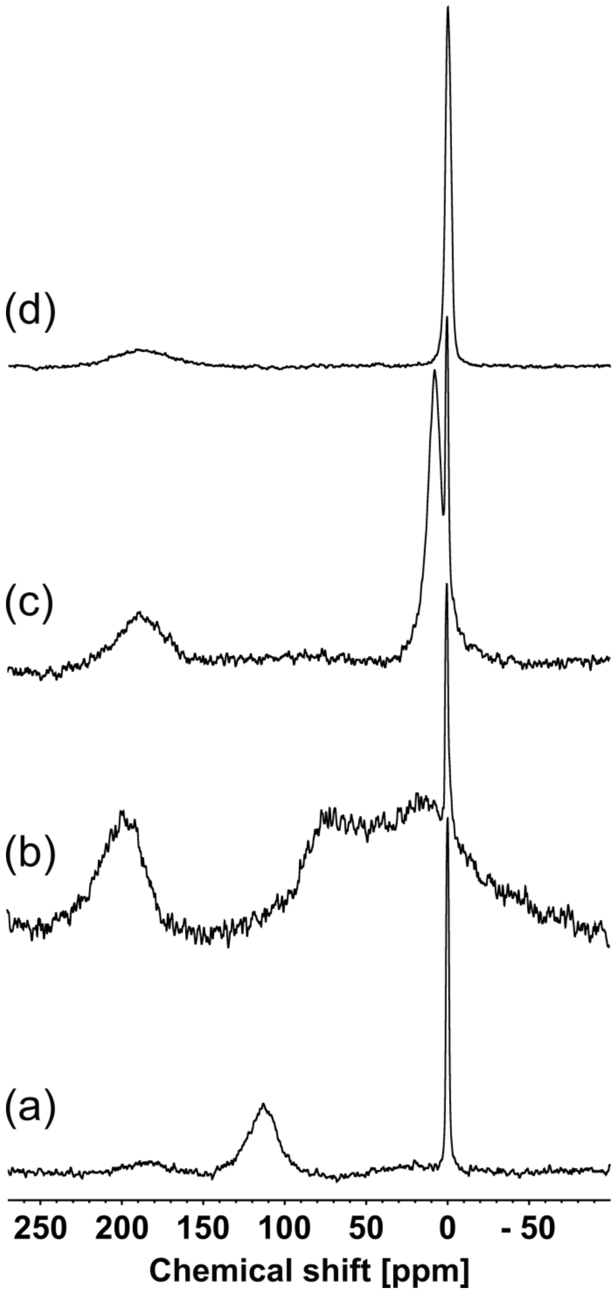


**Figure S4.** $^{129}$Xe NMR spectra of (a) REF 1, (b) REF 2, (c) REF 3 and (d) REF 4 acquired at 25 ˚C.

## 5. Room temperature $^{129}$Xe NMR spectrum of sample MIP 1 in the presence of bupivacaine

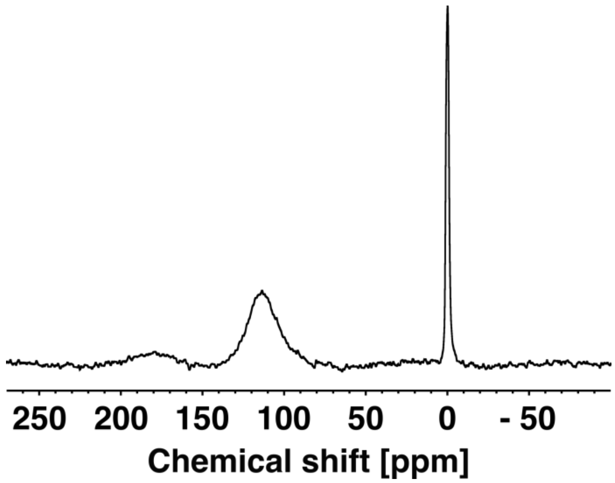


Figure S5. $^{129}$Xe NMR spectrum of sample MIP 1 in the presence of bupivacaine acquired at 25 °C.

## 6. Variable temperature $^{129}$Xe NMR all MIP and REF samples not presented in the main text, and temperature dependent plot of the chemical shift of the $\delta_2$ signal

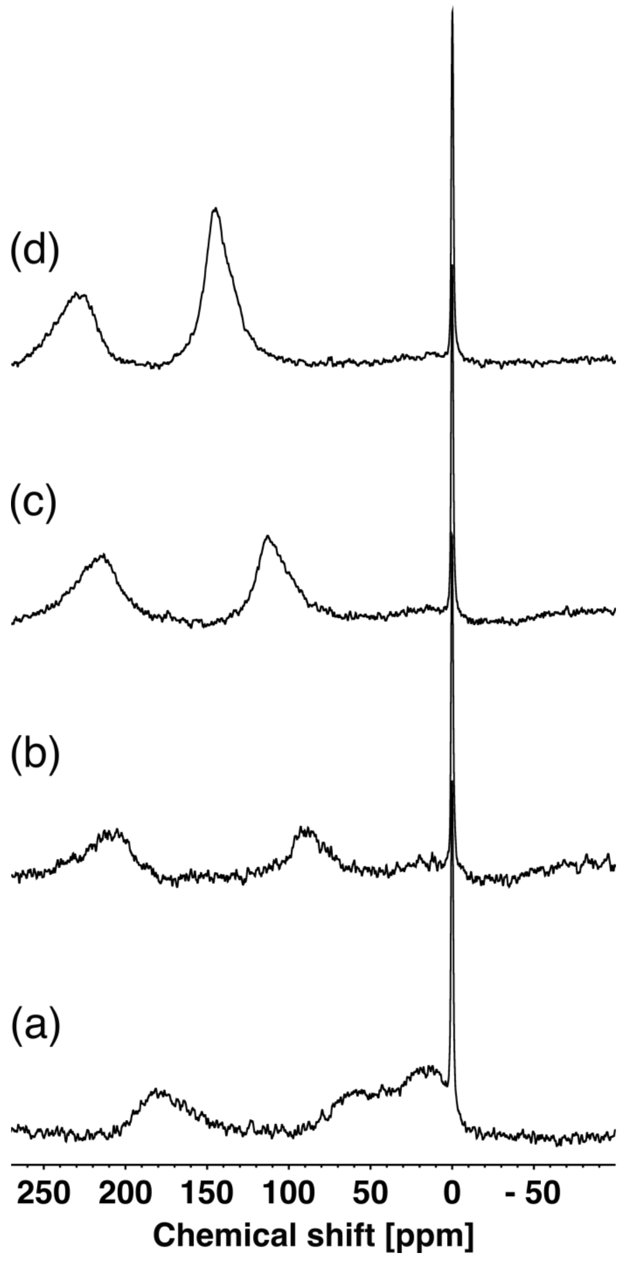

**Figure S6.** $^{129}Xe$ NMR spectra of sample MIP 2 acquired at (a) 25 ˚C, (b) 0 ˚C, (c) -20 ˚C, (d) -40 ˚C.

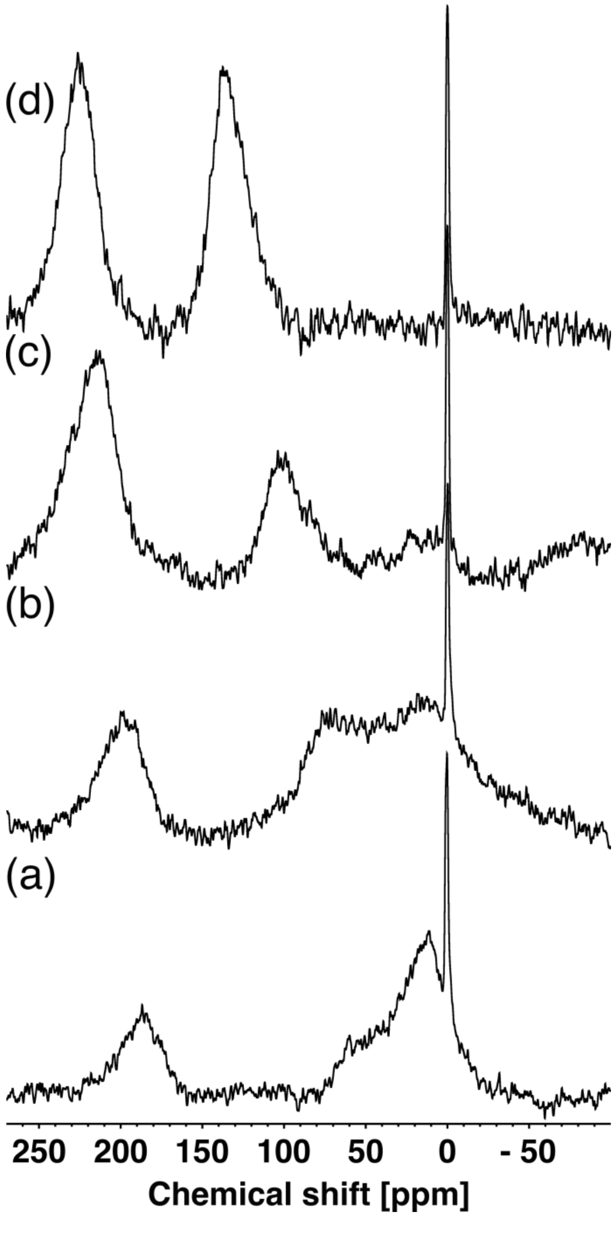


**Figure S7.** $^{129}Xe$ NMR spectra of sample REF 2 acquired at (a) 25 ˚C, (b) 0 ˚C, (c) -20 ˚C, (d) -40 ˚C.

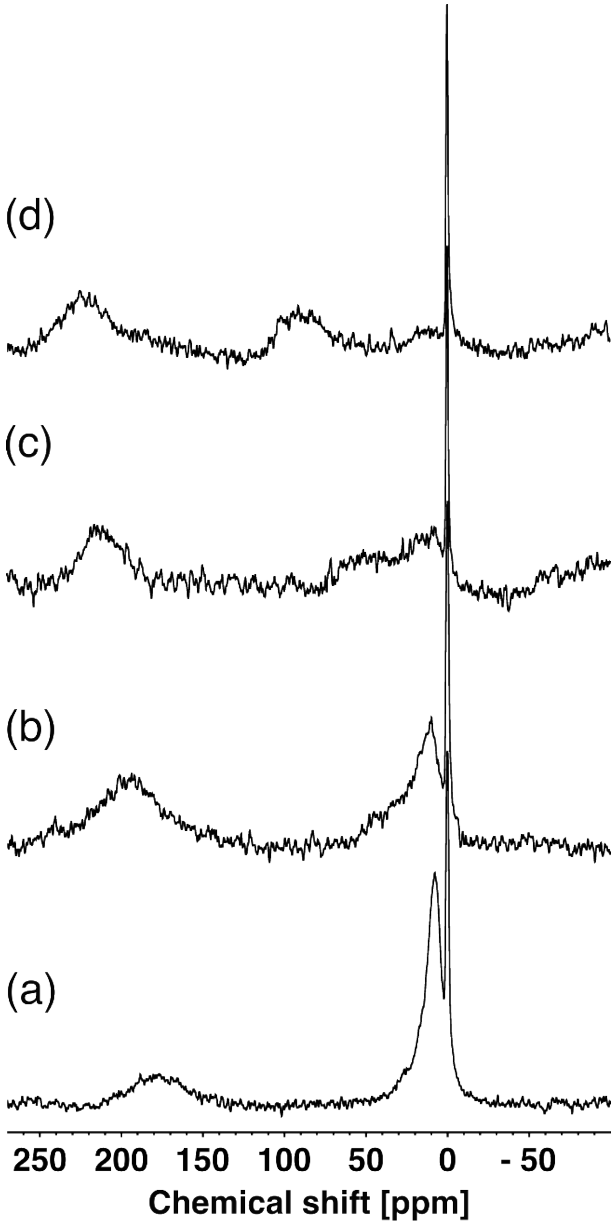


**Figure S8.** $^{129}Xe$ NMR spectra of sample MIP 3 acquired at (a) 25 ˚C, (b) 0 ˚C, (c) -20 ˚C, (d) -40 ˚C.

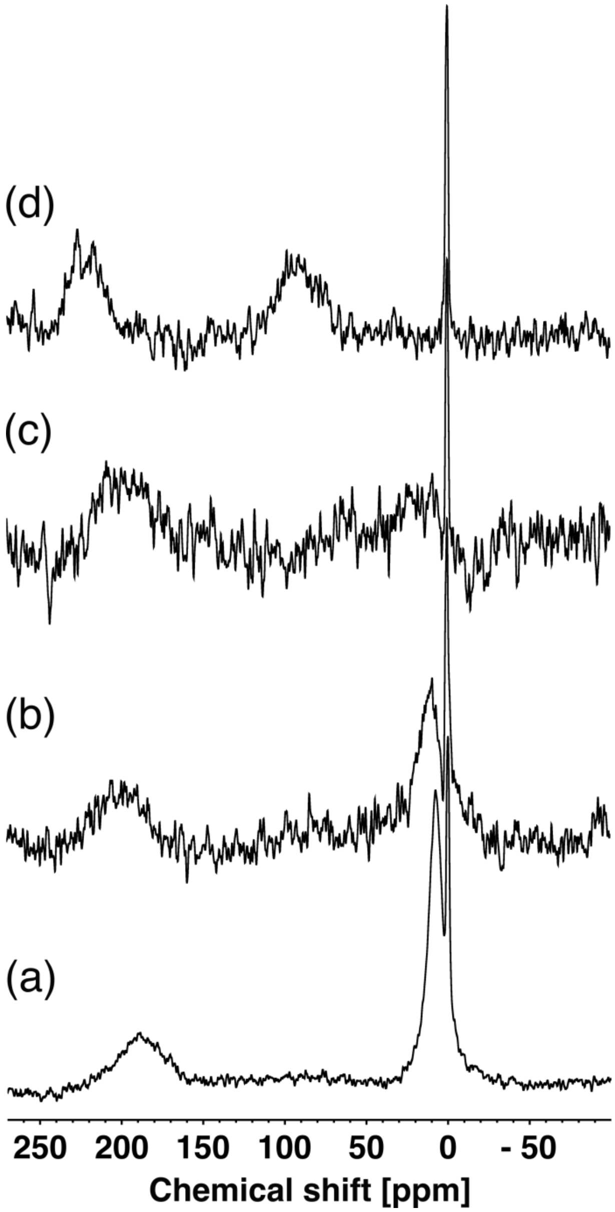


**Figure S9.** $^{129}Xe$ NMR spectra of sample REF 3 acquired at (a) 25 ˚C, (b) 0 ˚C, (c) -20 ˚C, (d) -40 ˚C.

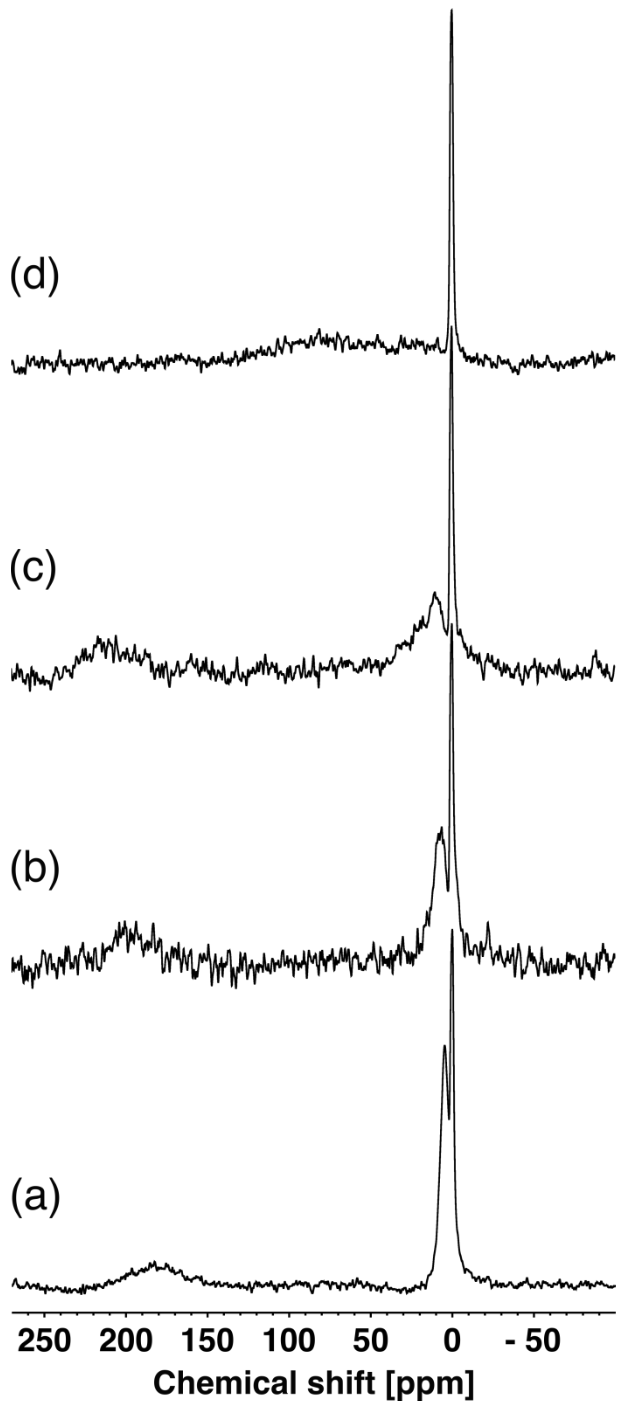


**Figure S10.** $^{129}Xe$ NMR spectra of sample MIP 4 acquired at (a) 25 ˚C, (b) 0 ˚C, (c) -20 ˚C, (d) -40 ˚C.

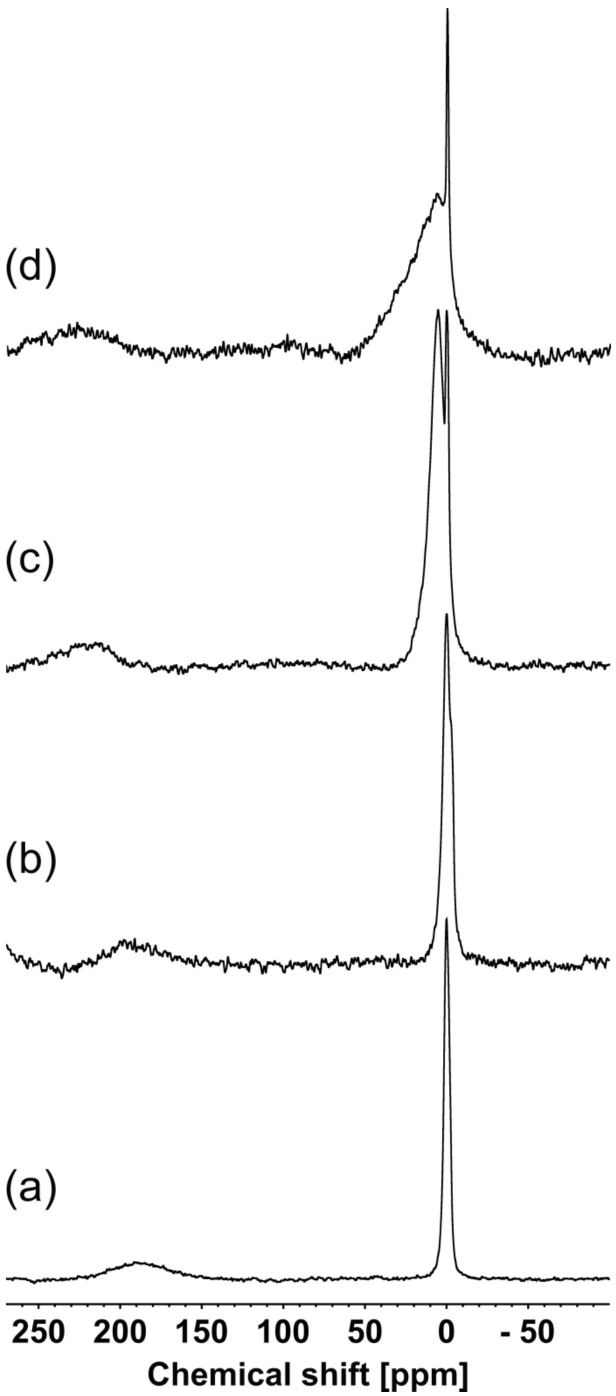


**Figure S11.** $^{129}$Xe NMR spectra of sample REF 4 acquired at (a) 25 ˚C, (b) 0 ˚C, (c) -20 ˚C, (d) -40 ˚C.

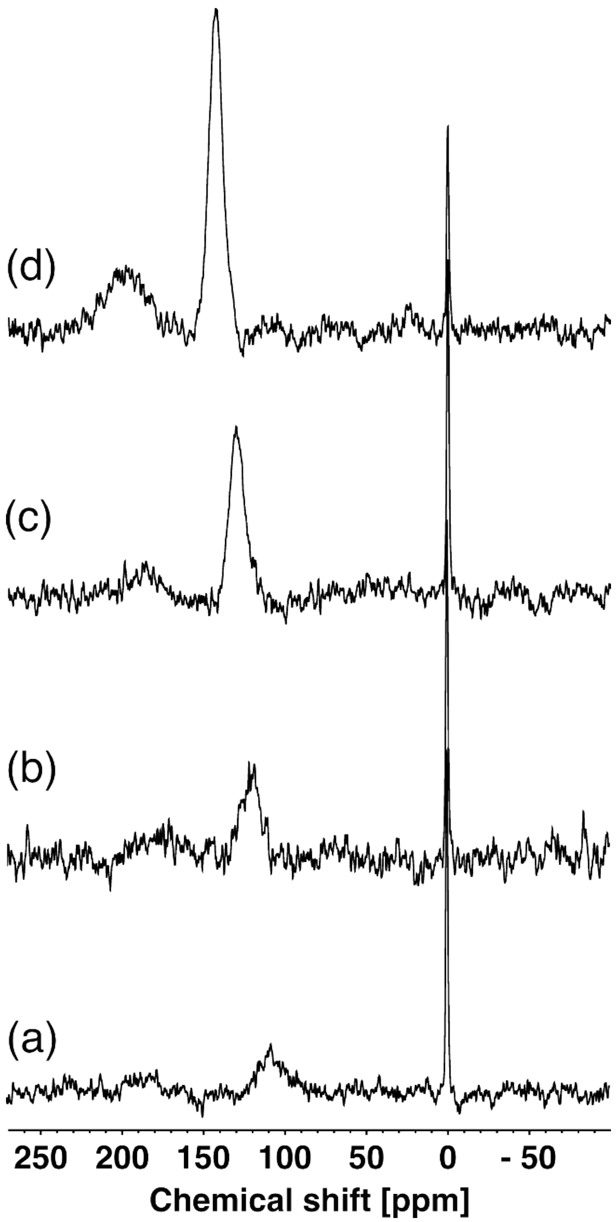


**Figure S12.** $^{129}Xe$ NMR spectra of sample MIP 1 with 1.4 bar nominal xenon pressure, acquired at (a) 25 ˚C, (b) 0 ˚C, (c) -20 ˚C, (d) -40 ˚C.

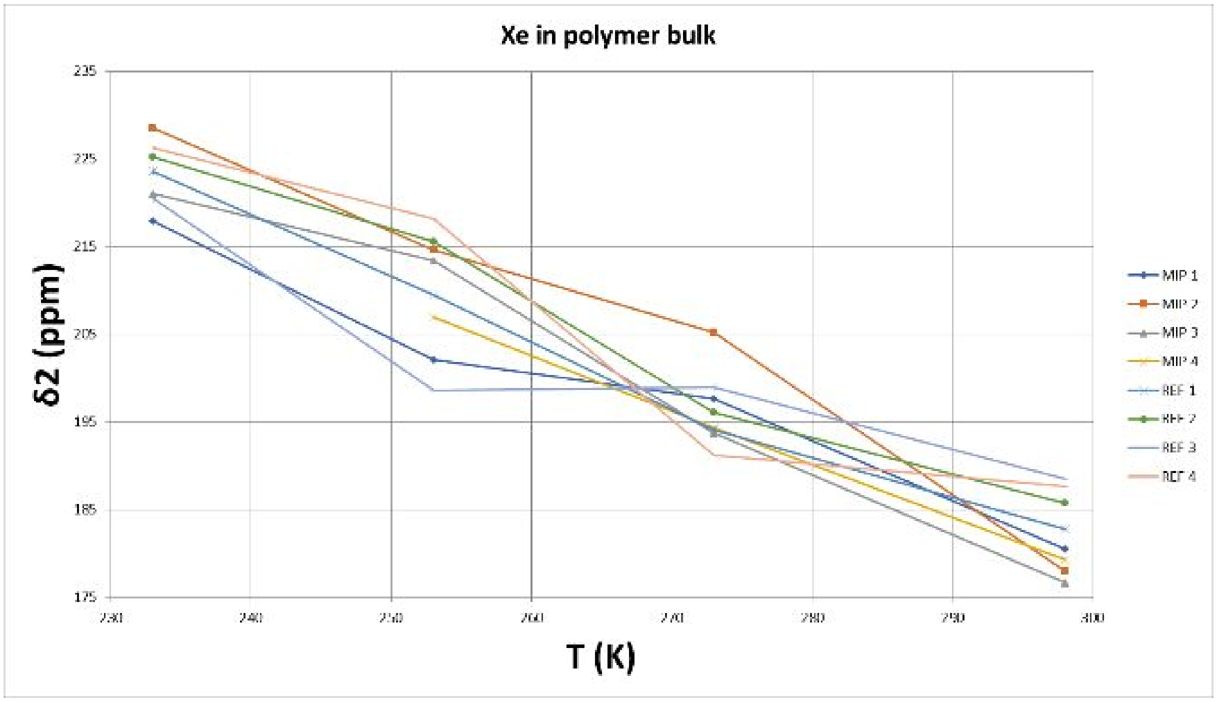


**Figure S13.** Temperature plot of signal $\delta_2$.
